# Rise in dermal CD11c^+^ dendritic cells associates with early-stage development of psoriatic lesions

**DOI:** 10.1007/s00403-012-1231-7

**Published:** 2012-03-22

**Authors:** Marcel B. M. Teunissen, Ling Zheng, Marjan de Groot, Menno A. de Rie, Jay S. Fine, Shu-Cheng Chen

**Affiliations:** 1 Department of Dermatology, Academic Medical Center, University of Amsterdam, M3-106, P.O. Box 22700, 1100 DE Amsterdam, The Netherlands; 2 Merck Research Lab, Legacy Schering-Plough Research Institute, K11-room 3025, BRID, 2000 Galloping Hill Road, Kenilworth, NJ 07033 USA; 3 Present Address: Boehringer Ingelheim Pharmaceuticals Inc., Ridgefield, CT USA

**Keywords:** Psoriasis, Point lesion, Early infiltrate, T cell, Dendritic cell, CD11c

## Abstract

There is limited information available regarding the phenotype and function of leukocytes involved in the earliest stages of psoriatic lesion development. In this study, we examined the presence of different types of leukocytes in psoriatic point lesions collected at three 1-week interval time points from a recent and simultaneously formed group of point lesions. The cells were quantified and compared with K16 expression and epidermal thickness, both typically increased in this disease and considered as hallmarks. We found a significant correlation between K16^+^ cell increment and the increase in epidermal thickness in the timeframe of 14 days. The change in CD3^+^, CD4^+^, and CD8^+^ T-cell numbers in the dermis showed a significant association with these two features from d7 to d14, whereas in the epidermis only CD8^+^ T cells demonstrated a significant correlation. Remarkably, the relationship between T cells and disease progression was preceded by a significant correlation of CD11c^+^ dendritic cells (DCs) with K16 expression and epidermal thickness from baseline onwards. Interestingly, there was also a numeric correlation of CD11c^+^ DCs with the CD3^+^ T-cell shifts from d7 to d14. A significant correlation was also found between dermal CD14^+^ cells and K16 expression from d7 to d14. BDCA-2^+^ plasmacytoid DCs were absent in non-lesional skin, but found at low numbers in most lesions. The change in plasmacytoid DC or neutrophil numbers did not correlate with lesion development. In conclusion, our study suggests a relevant role for T cells, and in particular dermal CD11c^+^ DCs, in the earliest stage of psoriatic lesion development.

## Introduction

Pronounced thickening of the epidermis with typical long rete ridges along with marked infiltrations of mononuclear cells in the papillary dermis are dominant aberrant histological features in psoriasis, but many more abnormalities can be observed in fully developed psoriatic plaques [13, 15, 24]. There are major alterations in the cellular constitution and extracellular matrix, the proliferation and differentiation of keratinocytes are affected, angiogenesis occurs, and also cells of innate and acquired immune system are activated. Despite tremendous efforts over the last decades to find the cause of psoriasis, the etiology of this inflammatory skin disease is still unknown, as it is hard to decide which features are relevant to the complex pathogenesis and which are just epiphenomenon.

To investigate the early onset of lesion, etiology is one approach to unravel the psoriatic pandemonium of multilevel, simultaneous and reciprocal interactions between multiple cell types. This approach can provide insight in the initial series of events that ultimately result in the formation of a full-blown lesion. The evolution of psoriatic lesions has been studied in various models, such as point lesions that newly appeared in symptomless skin [2, 6–8, 10, 16, 19], marginal zones of active psoriatic plaques with centrifugal expansion [26], Koebner reactions induced by traumatic injury in symptomless skin [1, 4, 9, 17], or reactivated lesions when relapse occurs after successful therapy [3, 23]. Symptomless skin from psoriasis patients transplanted onto immunodeficient mice also provides a useful model, as the transplant can convert (either spontaneously or induced) into lesional skin with psoriatic hallmarks [21, 22]. In this study, we have focused on changes in the presence and distribution of various leukocyte types in incipient point lesions to estimate alterations that correlate significantly with the early-stage development of psoriasis.

## Materials and methods

### Patients and biopsies

Six patients (25–79 years, mean 52 years; 4 men, 2 women) with plaque psoriasis were included, who did not receive any systemic or topical treatment (for 4 and 2 weeks, respectively) prior to and during the study. This study was approved by the institutional ethical committee and all patients gave informed consent before participation. In each patient, a skin area with several, close-ranged (approximately 2 cm distance), newly developed point lesions was selected. One 3-mm biopsy was taken from one point lesion at baseline (d0) and from adjacent point lesions at d7 and d14. In five patients a biopsy was taken from symptomless skin. Biopsy samples were snap-frozen in OCT compound and stored at −80 °C.

### Antibodies and immunohistochemistry

We have used antibodies against: K16 (Novocastra Laboratories Ltd, New Castle, UK), CD11c (BD Pharmingen, San Diego, CA), CD1a, CD3, CD4, and CD8 and CD14 (Dako Cytomation, Carpinteria, CA), BDCA-2 (Miltenyi Biotec, Auburn, CA), and CD66b (BioLegend, San Diego, CA). The staining procedure was carried out as reported previously [5].

### Digital image and histomorphometric analysis

For each section, overlapping adjacent fields of images were acquired at a 200× magnification with a digital camera and a motorized Eclipse 90i microscopy system equipped with NIS-Elements image analysis program (Nikon Instruments Inc., Melville, NY) and integrated into one large image. The resultant color images used in the following quantification analysis were in a 1,280 × 1,024 pixel RGB format with a 64-bit resolution. For each marker, the microscope and the camera were calibrated according to a standardized procedure and these settings were used for all biopsies.

The K16^+^ stained area and total epidermal area were measured. The percentage of K16^+^ epidermal area was used in the correlation analysis. For the other stainings, positive cells were manually counted in the epidermis and dermis separately. The numbers of positive cells were expressed as the number of positive cells per square millimeter (mm^2^) of epidermis or dermis. The average epidermal thickness was determined by taking the average epidermal thickness from five different areas of each section measured with NIS-Element program.

### Statistical analysis

The non-parametric Spearman’s correlation (SPSS software) was used to assess the statistical difference of various parameters between d0, d7 and d14, and *p* values of 0.05 or less were considered significant.

## Results

### Characterization of epidermal changes

**Fig. 1:**
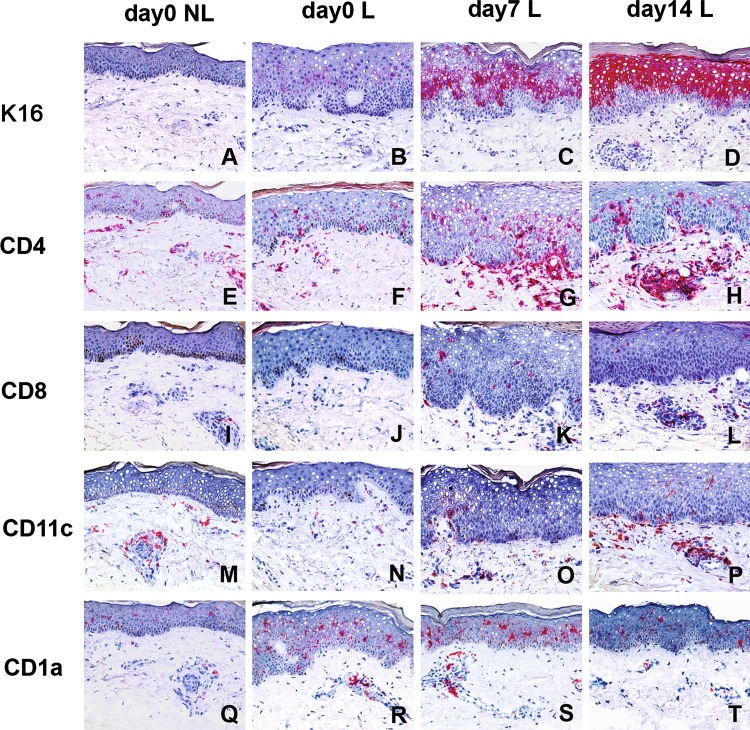
Expression of K16, CD4, CD8, CD11c and CD1a in early-stage psoriatic lesions. Biopsies were taken from non-lesional skin (day0 NL) and from incipient psoriatic lesions at baseline (day0 L) and 1 (day7 L) and 2 (day14 L) weeks later. Cryostat sections were stained by means of immunohistochemistry for keratinocyte differentiation marker K16 (**a**–**d**) and for the presence of CD4 (**e**–**h**) and CD8 (**i**–**l**), CD11c (**m**–**p**) and CD1a (**q**–**t**). Positively stained cells are in *red* color

Six psoriasis patients were selected having recently formed point lesions that had simultaneously developed in a close-ranged area. Biopsies were taken at baseline and at time points d7 and d14. Histological evaluation revealed epidermal hyperplasia in all six baseline lesional biopsies as compared to non-lesional samples taken from the same patients. Keratinocyte differentiation marker K16 was examined to assess the activation state of the epidermis [11]. For all six cases, epidermal thickness and K16 expression showed a tendency to increase from d0 to d7 (Fig. 1b, c). From d7 to d14, the levels of both parameters were either sustained or increased in three biopsies (Fig. 1c, d), while the remaining three cases showed an unexpected decline (incongruous with the clinical appearance) in both histological parameters (data not shown). There was a significant correlation between K16 expression and the change of epidermal thickness in all six cases between d0 and d7 as well as d7 and d14 (Table 1).

**Table 1:** Correlation of K16, epidermal thickness and leukocyte markers in early-stage psoriasis lesions

Cell markers	Anatomical sites	Correlations	∆(d7–d0)		∆(d14–d7)	
			*r*	*p*	*r*	*p*
K16	Epidermis	vs. **∆** Epi thickness	**0.943**	**0.005**	**0.943**	**0.005**
CD3	Epidermis	vs. **∆** Epi thickness	0.371	0.468	0.771	0.072
		vs. **∆** K16 cells	0.2	0.704	**0.829**	**0.042**
	Dermis	vs. **∆** Epi thickness	0.429	0.397	**0.886**	**0.019**
		vs. **∆** K16 cells	0.657	0.156	**0.943**	**0.005**
CD4	Epidermis	vs. **∆** Epi thickness	0.257	0.623	0.371	0.468
		vs. **∆** K16 cells	0.371	0.468	0.257	0.623
	Dermis	vs. **∆** Epi thickness	0.314	0.544	**0.886**	**0.019**
		vs. **∆** K16 cells	0.486	0.329	**0.943**	**0.005**
CD8	Epidermis	vs. **∆** Epi thickness	0.6	0.208	**0.829**	**0.042**
		vs. **∆** K16 cells	0.657	0.156	0.771	0.072
	Dermis	vs. **∆** Epi thickness	0.657	0.156	**0.943**	**0.005**
		vs. **∆** K16 cells	0.429	0.397	**1**	**<0.001**
CD11c	Epidermis	vs. **∆** Epi thickness	0.2	0.704	0.6	0.208
		vs. **∆** K16 cells	0.371	0.468	0.714	0.111
		vs. **∆** CD3 cells	0.486	0.329	**0.943**	**0.005**
	Dermis	vs. **∆** Epi thickness	**0.943**	**0.005**	**0.943**	**0.005**
		vs. **∆** K16 cells	**0.886**	**0.019**	**1**	**<0.001**
		vs. **∆** CD3 cells	0.371	0.468	**0.943**	**0.005**
CD1a	Epidermis	vs. **∆** Epi thickness	0.086	0.872	**−0.886**	**0.019**
		vs. **∆** K16 cells	0.029	0.957	−0.771	0.072
	Dermis	vs. **∆** Epi thickness	0.657	0.156	0.429	0.397
		vs. **∆** K16 cells	0.486	0.329	0.6	0.208
CD14	Epidermis	vs. **∆** Epi thickness	−0.091	0.864	0.516	0.295
		vs. **∆** K16 cells	−0.03	0.95	0.698	0.123
	Dermis	vs. **∆** Epi thickness	0.429	0.397	0.771	0.072
		vs. **∆** K16 cells	0.657	0.156	**0.829**	**0.042**
BDCA-2	Dermis	vs. **∆** Epi thickness	0.771	0.072	0.6	0.208
		vs. **∆** K16 cells	0.543	0.266	0.657	0.156

Markers indicated in the first column are compared with changes (vs. **∆**) in epidermal thickness (Epi thickness), K16^+^ keratinocyte (ΔK16 cells) or CD3^+^ T cell numbers (ΔCD3 cells) from d0 to d7 [Δ(d7–d0)] or d7 to d14 [Δ(d14–d7)]. All other combination of markers that are not indicated in this table did not reach significant correlation

Statistic significant correlations are depicted in bold. *r* = correlation factor, *p* = value of significance

### The number of T cells associated with the severity of the psoriatic lesions

Similar to non-lesional skin, CD4^+^ T cells showed a scattered distribution in the epidermal and dermal area in the early-stage psoriatic lesion at baseline (Fig. 1e vs. f), while CD8^+^ T cells were sparse in the epidermis and rarely found in dermis (Fig. 1i vs. j). Although at d7, clearly increased numbers of epidermal and dermal CD4^+^ and CD8^+^ T cells were observed in all six biopsies as compared to d0 (Figs. 1f vs. g, j vs. k, 2), there was no significant correlation found between the increase of T cells and changes in K16 expression or epidermal thickness in this time period (Table 1). Between d7 and d14, a significant correlation was found between the alteration in dermal CD3^+^, CD4^+^ or CD8^+^ T cells and changes in epidermal K16 expression or epidermal thickness (Table 1). In addition, changes in epidermal CD3^+^ T cells were also found to correlate with changes in K16 expression, whereas changes in epidermal CD8^+^ T cells correlated with epidermal thickness (Table 1).

**Fig. 2:**
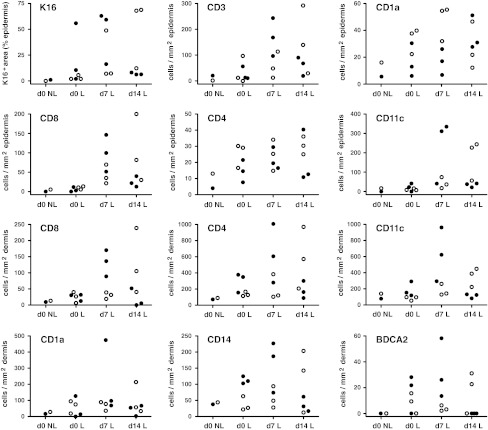
Count for K16 and leukocytes in early-stage psoriatic lesions. Biopsies were taken from non-lesional skin (d0 NL) and incipient psoriatic lesions at baseline and 1 and 2 weeks later (d0 L, d7 L, d14 L, respectively). Cryostat sections were stained for K16 expression and the presence of CD3^+^, CD4^+^, and CD8^+^ T cells, CD1a^+^ Langerhans cells and dendritic cells, CD11c^+^ dendritic cells, CD14^+^ macrophages and BDCA-2^+^ plasmacytoid dendritic cells. The *two top rows* are countings of the epidermis while the *bottom two rows* are dermal cell numbers. *Closed symbols* represent cell numbers of the three cases that showed a decline of epidermal thickness and K16 expression from d7 to d14, while the *open symbols* represent the three cases that showed increment of epidermal thickness and K16 expression from d0 to d14. Statistics of all these numbers are depicted in Table 1

### Dermal CD11c^+^ dendritic cell increment associated with psoriatic lesion progression

At baseline, CD11c^+^ dendritic cells (DCs) were distributed predominantly in the dermal perivascular area, in a similar pattern as in non-lesional skin (Fig. 1m vs. n). The number of CD11c^+^ DCs was consistently increased (especially outside the perivascular area) in the three cases in which the lesion development proceeded from d0 to d14 (Figs. 1n–p, 2). The number of CD11c^+^ DCs declined from d7 to d14 in the three cases that showed a decline of epidermal thickness and K16 expression from d7 to d14 (data not shown). The shift in dermal CD11c^+^ DC numbers was the only parameter that showed significant correlation with psoriatic lesion development from d0 through d14 as measured by both epidermal thickness and K16 expression (Table 1). Remarkably, the change in CD11c^+^ DC numbers also correlated significantly with the changes in numbers of dermal and epidermal CD3^+^ T cells from d7 to d14 (Table 1).

The number of epidermal CD1a^+^ cells in d0 new lesions was higher (not significant) than in non-lesional baseline samples (Fig. 1q–t). From d0 to d14, there were variable non-significant alterations in the number of epidermal and dermal CD1a^+^ cells in new lesions (Fig. 2). From d7 to d14, a significant inverse correlation was found between epidermal CD1a^+^ cells and epidermal thickness (Table 1). BDCA-2^+^ plasmacytoid DCs (PDCs) were undetectable in non-lesional skin (Fig. 2). Small numbers of BDCA-2^+^ PDCs were seen in most early-stage psoriatic lesions, mainly in the epidermal–dermal junction, from d0 to d14 without obvious differences between these time points. No correlations were found between BDCA-2^+^ PDCs and epidermal thickness or K16 expression in this time course. In the early-stage psoriatic lesion at baseline, CD14^+^ macrophages were only detected in the dermal area, as was the case for non-lesional skin. The change in CD14^+^ cell numbers seen in the dermis between d7 and d14 was correlating with the K16 expression (Table 1; Fig. 2). An occasional or no CD14^+^ cell was present in the epidermis in the 14 days period (data not shown). Only very few CD66b^+^ neutrophils were found in the dermis and epidermis in some early lesions revealing non-significant differences between the time points (data not shown).

## Discussion

We have studied the presence and distribution of various leukocyte types in recently arisen psoriatic point lesions to estimate which cells are involved in the onset of psoriatic plaque formation. The numeric changes in leukocyte populations were compared with changes in epidermal thickness and K16 expression, which are two typical features that represent the activation state of the psoriatic epidermis [1], and which nicely correlated from baseline to d14 in all our incipient point lesions. The rise in dermal CD11c^+^ DC numbers was the only change that correlated significantly with the intensification of these two features during the earliest test period d0–d7. Moreover, in the next period (d7–d14), the significant correlation between alterations of dermal CD11c^+^ DC numbers and activation state of the epidermis remained; i.e., the number of DCs raised along with the increment of epidermal thickness and K16 expression in three patients, and visa versa, the number of dermal CD11c^+^ DCs decreased in the other three patients who showed an unexpected decline in epidermal hyperplasia. One explanation for this decline in epidermal hyperplasia could be spontaneous remission. In the period d7–d14, the shift in dermal CD11c^+^ DC numbers also achieved significant correlation with the changes in T-cell numbers. Although T cells are generally regarded to be essentially involved in the pathogenesis of psoriasis [13, 15, 24], correlation of the shift in T cell numbers with epidermal thickness and K16 expression was not significant in the earliest test period d0–d7, but dermal CD4^+^ and CD8^+^ T cells reached significant correlation somewhat later during d7–d14. The inverse correlation of epidermal CD1a^+^ cells with epidermal hyperplasia during d7–d14 is likely due to the relative rapid-expanding epidermal surrounding compared to CD1a^+^ cell numbers. A decrease of CD1a^+^ cells in early psoriatic lesions has also been found by others [19], whereas more numerous CD1a^+^ cells can be found in the chronic phase [16]. Altogether, the influx of CD11c^+^ DCs in the dermis showed the most prominent correlation with the earliest development of psoriatic lesions.

Various studies on recently formed psoriatic lesions have been performed in the pre-immunochemistry era and were based on hematoxylin and eosin or enzyme staining. The results in these studies were rather conflicting, as some reported that the early cellular infiltrate mainly comprises numerous polymorphonuclear cells [6, 9], whereas in line with our results, others [2, 10] reported that the early infiltrate is mainly mononuclear along with rare occurrence of polymorphonuclear cells. From the 1980s, when antibodies became available for immunohistochemistry, the studies on recently formed psoriatic lesions were primarily focused on CD4^+^ and CD8^+^ T cells and revealed that CD4^+^ T cells predominated in the dermis and epidermis in the earliest phase later followed by preponderance of CD8^+^ T cells in the epidermis [8, 16, 17, 19]. Our results not only confirm that the infiltration of CD4^+^ and CD8^+^ T cells correlates with the development of psoriatic lesions, but also demonstrate that this elevation in T cells is preceded by an influx of CD11c^+^ DCs in the dermis. Although PDCs have been described to be involved in the early development of psoriasis [14], we could only demonstrate the presence of these cells in incipient lesions, but could not find a correlation with the early development of psoriatic lesions. This lack of correlation is most likely due to the limited number of patients.

Under pathological conditions, the skin is infiltrated by several different subsets of inflammatory DCs [25], among others the so-called CD11c^+^BDCA-1^−^ Tip-DCs [12]. The Tip-DCs have a relevant contribution to the development and maintenance of psoriatic lesions as they are not only able to produce proinflammatory and immunomodulatory factors, like TNF-α and inducible nitric oxide synthase, IL-20 and IL-23, but also capable of polarizing T-cell activation towards Th1/Th17 [12, 18, 28]. In addition, the CD11c^+^ DC population may also directly promote epidermal hyperplasia as they produce large amounts of IL-22 [20]. Moreover, the earliest signs (within 1–2 weeks) of successful therapy with etanercept comprise the reduction of multiple inflammatory products of Tip-DCs [27].

In summary, we have demonstrated that the earliest development of psoriatic lesions most markedly correlated with the increment of CD11c^+^ DCs in the dermis. This suggests that these cells have a relevant role in the generation of psoriatic lesions and that CD11c^+^ DCs may serve as target for therapy.
